# Linking coupled motions and entropic effects to the catalytic activity of 2-deoxyribose-5-phosphate aldolase (DERA)†
†Electronic supplementary information (ESI) available: Further experimental and computational data. See DOI: 10.1039/c5sc03666f


**DOI:** 10.1039/c5sc03666f

**Published:** 2015-11-17

**Authors:** Huan Ma, Klaudia Szeler, Shina C. L. Kamerlin, Mikael Widersten

**Affiliations:** a Department of Chemistry – BMC , Uppsala University , Box 576 , SE-751 23 Uppsala , Sweden . Email: mikael.widersten@kemi.uu.SE; b Department of Cell and Molecular Biology , Uppsala University , Box 596 , SE-751 24 , Uppsala , Sweden . Email: kamerlin@icm.uu.SE

## Abstract

DERA, 2-deoxyribose-5-phosphate aldolase, catalyzes the retro-aldol cleavage of 2-deoxy-ribose-5-phosphate (dR5P) into glyceraldehyde-3-phosphate (G3P) and acetaldehyde in a branch of the pentose phosphate pathway. In addition to the physiological reaction, DERA also catalyzes the reverse addition reaction and, hence, is an interesting candidate for bio-catalysis of carbo-ligation reactions, which are central to synthetic chemistry. An obstacle to overcome for this enzyme to become a truly useful biocatalyst, however, is to relax the very strict dependency of this enzyme on phosphorylated substrates. We have studied herein the role of the non-canonical phosphate-binding site of this enzyme, consisting of Ser^238^ and Ser^239^, by site-directed and site-saturation mutagenesis, coupled to kinetic analysis of mutants. In addition, we have performed molecular dynamics simulations on the wild-type and four mutant enzymes, to analyse how mutations at this phosphate-binding site may affect the protein structure and dynamics. Further examination of the S239P mutant revealed that this variant increases the enthalpy change at the transition state, relative to the wild-type enzyme, but concomitant loss in entropy causes an overall relative loss in the TS free energy change. This entropy loss, as measured by the temperature dependence of catalysed rates, was mirrored in both a drastic loss in dynamics of the enzyme, which contributes to phosphate binding, as well as an overall loss in anti-correlated motions distributed over the entire protein. Our combined data suggests that the degree of anticorrelated motions within the DERA structure is coupled to catalytic efficiency in the DERA-catalyzed retro-aldol cleavage reaction, and can be manipulated for engineering purposes.

## Abstract 2

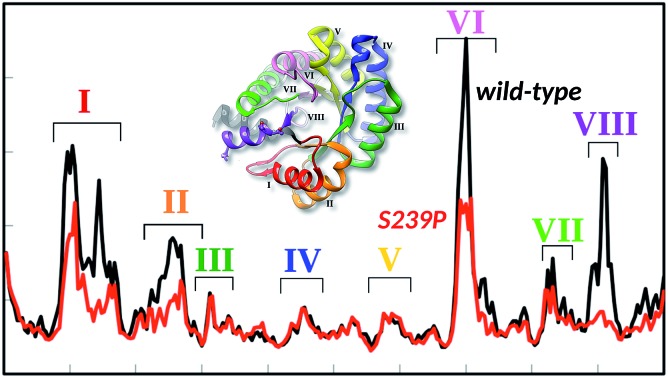
Local mutations in the phosphate binding group of DERA alter global conformation dynamics, catalytic activities and reaction entropies.

## Introduction

Aldol addition is a central reaction type in both synthetic chemistry and cellular metabolism, and is the most commonly applied reaction for the synthesis of poly-hydroxylated compounds with new chiral centers.^1^ Aldehydes are of particular interest as donor molecules in these addition reactions, due to the fact that they form other aldehydes as products that can be readily subjected to further addition reactions leading to increasingly complex structures.^2^*In vivo*, aldolases catalyze the stereoselective aldol addition of aldehydes and ketones or the cleavage of the corresponding aldols.

**Fig. 1:**
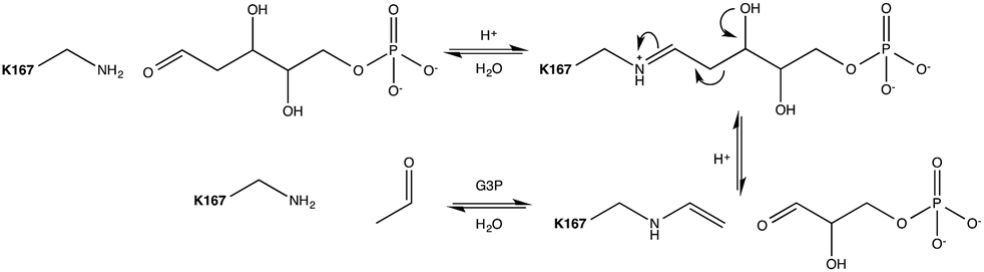
Mechanism of the DERA catalysed aldol cleavage of dR5P. The side-chain amine of Lys^167^ condenses with the incoming aldose to form the imine equivalent. The imine facilitates C–C bond cleavage between carbons 2 and 3 by resonance stabilization of the resulting carbanion. The Schiff base stabilizes the enamine formed by either the catalysed cleavage of an aldol to the respective components, or by the deprotonation of the α-proton of a donor aldimine (or ketimine) during the aldol addition reaction. The catalytic cycle is completed by hydrolysis of the enamine. Auxiliary catalytic residues have been omitted for clarity.

More than 30 different aldolases have been described to date, which are in turn classified into two major classes according to their catalytic mechanisms.^3^ Class I aldolases such as the enzyme of interest to this work activate the donor molecule by replacing the carbonyl oxygen with a nitrogen from an active-site Lys residue, thus forming an imine Schiff base as a reaction intermediate (Fig. 1). Note that the stereochemistry of the newly formed stereocentre is controlled by the enzyme regardless of the structure of the reactant substrate, which facilitates predictions of the product's stereo-configuration. These enzymes have attracted great attention as potential environmentally friendly alternatives for the catalysis of carboligation reactions, and much effort is being invested into understanding their structure–activity relationships, and their putative roles as greener catalysts in organic synthesis.^4^

**Fig. 2:**
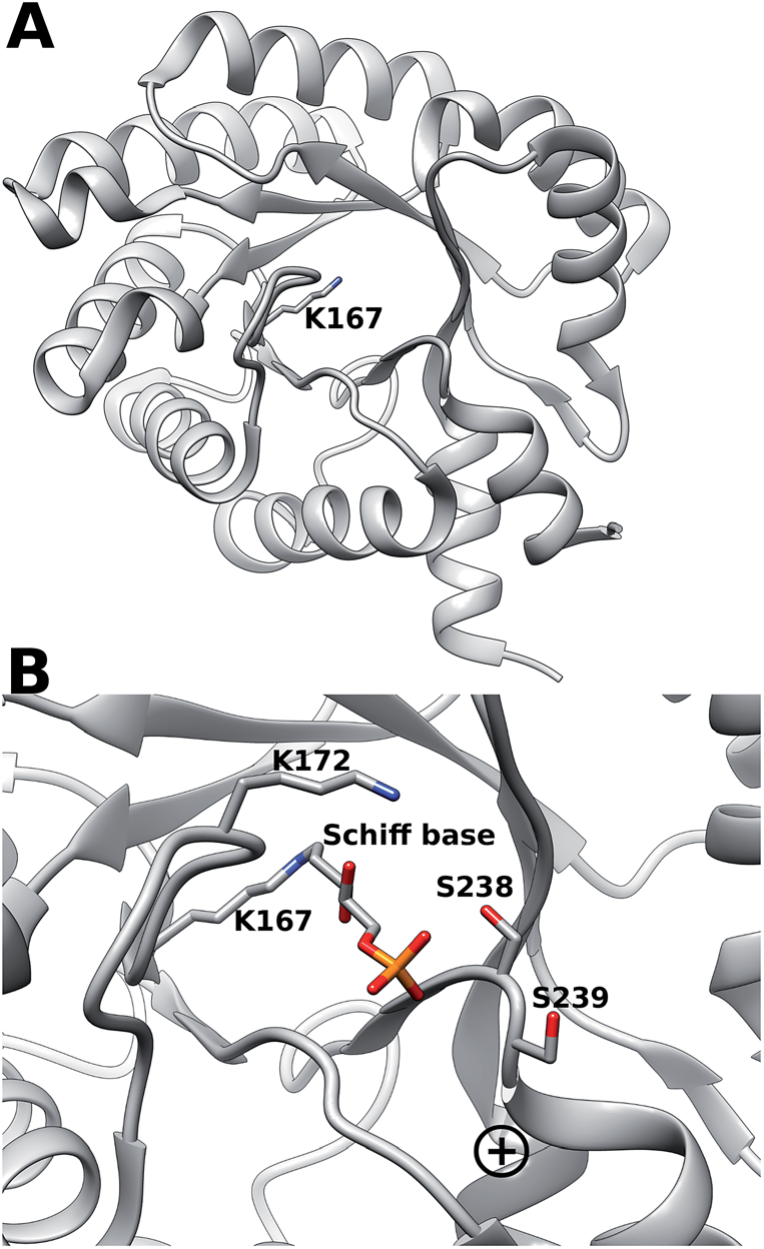
(A) (α/β)_8_ TIM barrel structure of DERA, highlighting the active-site Lys^167^ that forms the Schiff base with the incoming aldehyde (dR5P or acetaldehyde). (B) Close-up of the DERA active site, with the Lys^167^–dR5P complex shown in a stick representation. The side-chains of Ser^238^ (two conformers in the crystal structure) and Ser^239^ and Lys^172^ are shown in sticks. The “plus” indicates the location of the N-terminus of α-helix 8. Image created with Chimera^6^ using the atomic coordinates in PDB ID ; 1JCJ.^7^,^8^

*Escherichia coli* 2-deoxyribose-5-phosphate aldolase (DERA, E.C. 4.1.2.4) is the only known Class I acetaldehyde dependent aldolase, and one out of only two known aldolases that catalyze the addition of two aldehydes.^5^ DERA is a 28 kDa monomeric protein consisting of 259 amino acid residues. It is a key enzyme in the pentose phosphate pathway of *E. coli*, as it catalyzes the reversible cleavage of d-2-deoxyribose-5-phosphate (dR5P) to d-glyceraldehyde-3-phosphate (G3P) and acetaldehyde. DERA displays a typical TIM (α/β)_8_ barrel fold, which is a common structural feature among the members of the Class I aldolase family (Fig. 2A).^7^ In DERA, the nucleophilic ε-amine of Lys^167^ located on the β6 strand, attacks the aldehyde functionality of the incoming substrate (acetaldehyde or dR5P), forming the corresponding imine Schiff base. If the substrate is dR5P, the reaction will proceed as outlined in Fig. 1 with C–C bond breaking and ultimate release of the products G3P and acetaldehyde. The rate-limiting step of the aldol cleavage has not been pinpointed, but is assumed to involve C–C bond breaking, which is in turn facilitated by the rearrangement of the formed carbanion to the stabilized enamine.

DERA is strictly dependent on phosphorylated acceptor substrates, which is illustrated by the drastic loss of activity when comparing G3P and d-glyceraldehyde as acceptor substrates.^5^ The main phosphate binding residues are Lys^172^ (*via* a bridging water molecule), as well as direct side-chain interactions with Ser^238^, direct peptide backbone interactions with Ser^238^ and Gly^205^, and water bridge interactions with the backbone moieties of Gly^171^, Val^206^, Gly^236^ and Ser^239^ (Fig. 2B). The Ser^238^–Ser^239^ motif located close to the C-terminal is an atypical phosphate-binding motif, and is not conserved among other Class I aldolases.^9^ Finally, a putative conformational change has been proposed to take place in this region upon substrate binding.^9^

**Fig. 3:**
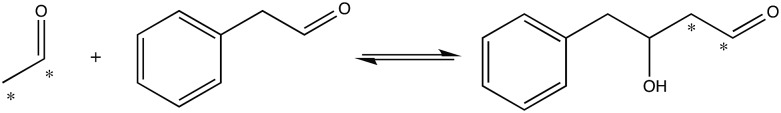
Reaction used to screen for S238X/S239X DERA variants with apparent activity towards an aryl-substituted aldehyde. Asterisks indicate ^14^C-label.

To investigate the importance of the phosphate binding site in DERA, we conducted mutagenesis of the Ser^238^/Ser^239^ pair by two approaches, firstly replacing these serine residues for prolines, and secondly introducing 11 other substitutions at either position using the NDT codon set. The resulting mutants were screened for catalysis of the reaction shown in Fig. 3. The rationale for the Pro replacements was to slightly move the position of the peptide backbone and thereby affect both side-chain interactions and backbone interactions with the phosphate group of the substrate. The shift in the position of the backbone was also intended to slightly move the N-terminal end of α-helix 8, thereby decreasing its proposed additional contribution from its positive dipole moment that can otherwise attract the phosphate group^9^ (Fig. 2B). The resulting S238P and S239P single mutants and the S238P/S239P double mutant were subsequently analysed for retro-aldolase activity towards dR5P, and we also examined the temperature dependence of the wild-type and S239P variants of DERA, as well as performing molecular dynamics simulations on all mutants.

Recent years have seen significant interest in understanding potential links between enzyme dynamics and correlated motions with both catalysis^10^–^17^ and also enzyme promiscuity and functional evolution.^18^,^19^ Following from this, there has also been discussion of the relevance of manipulating such dynamics in artificial enzyme design.^20^–^22^ Our combined experimental and computational analysis of the dynamical behaviour of these DERA mutants strongly suggests a role for coupled motions and entropic changes in driving the catalytic activity of this enzyme, and that the detrimental changes in the activities of the mutants we studied can be linked to both a loss in correlated motions as well as changes in activation entropies. Such dynamical changes can, in turn, be used to modulate the activity of this biocatalytically important enzyme.

## Results and discussion

An overview of the kinetic parameters for the S238P and S239P single mutants, as well as the S238P/S239P double mutant is shown in Table 1. As can be seen from this table, both the S238P variant and the corresponding double mutant displayed completely abolished catalytic activity in the retro-aldol reaction with this substrate, which strongly suggests a crucial role for position 238 in catalysis, either because a Ser specifically is important at that position, or because the inclusion of Pro is detrimental. In contrast, in the case of Ser^239^, the main effect of this replacement seems to be merely to increase *K*dR5PM by about 30-fold, and this mutant appears to otherwise readily catalyse the cleavage of dR5P (Table 1). Therefore, Ser^239^ appears here to be less crucial for catalysis than Ser^238^.

**Table 1:** Measured kinetics of aldol cleavage by wild-type and mutant forms of DERA^*a*^

Enzyme	*k*_cat_ (s^–1^)	*k*_cat_/*K*_M_^*b*^ (s^–1^ M^–1^)	*K*_M_ ^*b*^ (M)
Wild type	15 ± 0.4	(1.3 ± 0.07) × 10^5^	(9.6 ± 0.8) × 10^–5^
S238P	—	<0.1	—
S239P	11 ± 1	(3.6 ± 0.06) × 10^3^	(3.0 ± 0.6) × 10^–3^
S238P/S239P	—	<0.1	—
S238I/S239I	1.4 ± 0.2	(1.5 ± 0.06) × 10^2^	(9.6 ± 1.5) × 10^–3^

^*a*^Reactions were assayed at pH 8 and at 30 °C.

^*b*^Calculated from the total concentration of dR5P.

Interpreting the precise origin of this change is complex, as in a multi-step reaction such as the DERA catalysed aldol cleavage, *K*_M_ is a composite constant that is built up of the rates for formation and decay of both the Michaelis complex of E·dR5P in both its furanose and open-chain forms, and also that of the formation and breakdown of the covalent Schiff base intermediate. Hence, destabilization of any of these enzyme-substrate species will increase the value of *K*dR5PM. Additionally, as the perturbed p*K*_a_ of the ammonium group of Lys^167^ ensures the presence of the nucleophilic amine at neutral pH, it is essential for catalytic activity. Therefore, if the acidity of this group was to be impaired by the S239P mutation, formation of the aldimine would also be impaired, which would also contribute to an increase in the value of *K*dR5PM. The pH dependencies of *k*_cat_ and *k*_cat_/*K*_M_ do not indicate this to be the case, however; the pH-rate profile of the S239P mutant is unchanged from that of wild-type DERA within the pH region assayed (Fig. S1†).

**Fig. 4:**
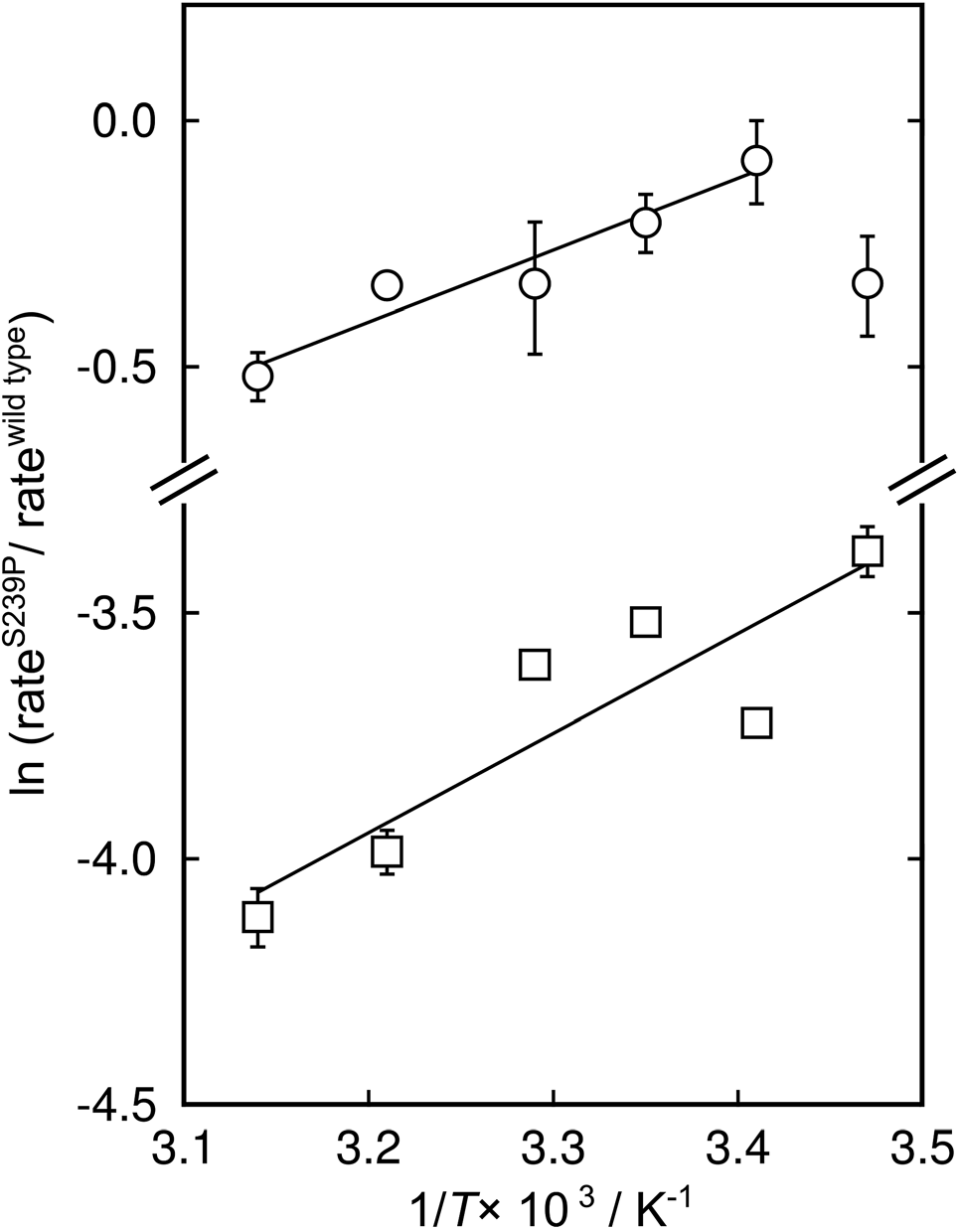
Modified Eyring plots of the effect by the S239P mutation on catalytic rates. The ratios of *k*_cat_ (circles) and *k*_cat_/*K*_M_ (squares) are plotted as a function of temperature. The slopes are described by –ΔΔ*H*^‡^/*RT*, and the intercepts by ΔΔ*S*^‡^/*R*. The extracted values of the differences in enthalpic and entropic parameters are given in Table 2.

There has been significant discussion about the role of entropy in enzyme catalysis (*e.g.*ref. 23–26). Therefore, the relative effects of the S239P mutation on activation enthalpies and entropies were also determined from the respective temperature dependencies of the catalysed rates (Fig. 4). As can be seen from Table 1, this mutation results in a ∼40-fold reduction of *k*_cat_/*K*_M_ compared to wild-type DERA. The thermodynamic data suggest that the main negative effect on catalysis caused by the S239P mutation is primarily a relative loss of activation entropy (Table 2). That is, the relative effects on the enthalpies are favourable, and of similar magnitude, for both the E·dR5P/E-dR5P → E + product(s) reaction, which would be represented by *k*_cat_, and the E + dR5P → E + product(s) reaction, which is reflected in *k*_cat_/*K*_M_. The observation that the mutation results in relative decreases in activation enthalpies is counter-intuitive, as one might assume that the phosphate interactions in the wild-type enzyme would contribute also to TS stabilization. We note, however, that since the values presented in Fig. 4 and Table 1 represent a double difference, it is not clear whether the main effect is on the transition state or the ground state, and it could equally be a ground state as a transition state effect.

**Table 2:** Effect of the S239P mutation on the thermodynamic parameters of the aldol cleavage reaction of dR5P

	ΔΔ*H*^‡^^*a*^ (kcal mol^–1^)	*T*ΔΔ*S*^‡^^*a*^ ^,^^*b*^ (kcal mol^–1^)	ΔΔ*G*^‡^^*c*^ (kcal mol^–1^)
*k*_cat_	–2.9 ± 0.5	–3.1 ± 0.5	0.19 ± 0.05
*k*_cat_/*K*_M_	–4.1 ± 1	–6.2± 1	2.2 ± 0.02

^*a*^Standard errors from linear least square regression.

^*b*^Calculated at 30 °C.

^*c*^Calculated at 30 °C from –*RT* ln(rate^S239P^/rate^wt^). The ΔΔ values represent the difference in each parameter between wild-type and mutant enzymes.

To further test the necessity of the presence of a *bona fide* phosphate binding site for the catalytic activity of DERA, as well as the possibility of adapting DERA into also accepting non-phosphorylated acceptor aldehydes, we performed limited saturation mutagenesis using the NDT codon set to replace Ser^238^ and Ser^239^. The resulting variants were assayed for their ability to incorporate ^14^C-labeled acetaldehyde into the acceptor phenyl acetaldehyde (Fig. 3). The assay is not quantitative but useful for screening purposes, and the reaction illustrated in Fig. 3 was specifically chosen based on both commercial availability of the arylsubstituted aldehyde, and the current industrial interest in producing biocatalysts that can catalyse aldol addition.^27^,^28^ Fig. S2† shows a typical result from a screen of aldol addition activities in bacterial lysates from DERA-variant expressing cells. Sequence analysis of the scored hits revealed that Ser was no longer over-represented in the hits with apparent aldol addition activity. Rather, Ile displayed the highest over-representation at position 238, followed by Ser in second and Val in third places (Fig. S3†). The order of preferred residues at position 239 was Leu > Ile > His. Ser was absent at position 239 in all picked hit variants. Since these activity screens had targeted the aldol addition of acetaldehyde and phenyl acetaldehyde it was not obvious whether these DERA variants would retain activity with phosphorylated substrates such as dR5P. To test this, an Ile^238^/Ile^239^ variant was purified and analyzed for activity with dR5P. This mutant did indeed exhibit reasonable activity also with this phosphorylated substrate (Table 1), which clearly demonstrates that the Ser^238^/Ser^239^ pair is not critical for catalytic activity in and of itself.

**Fig. 5:**
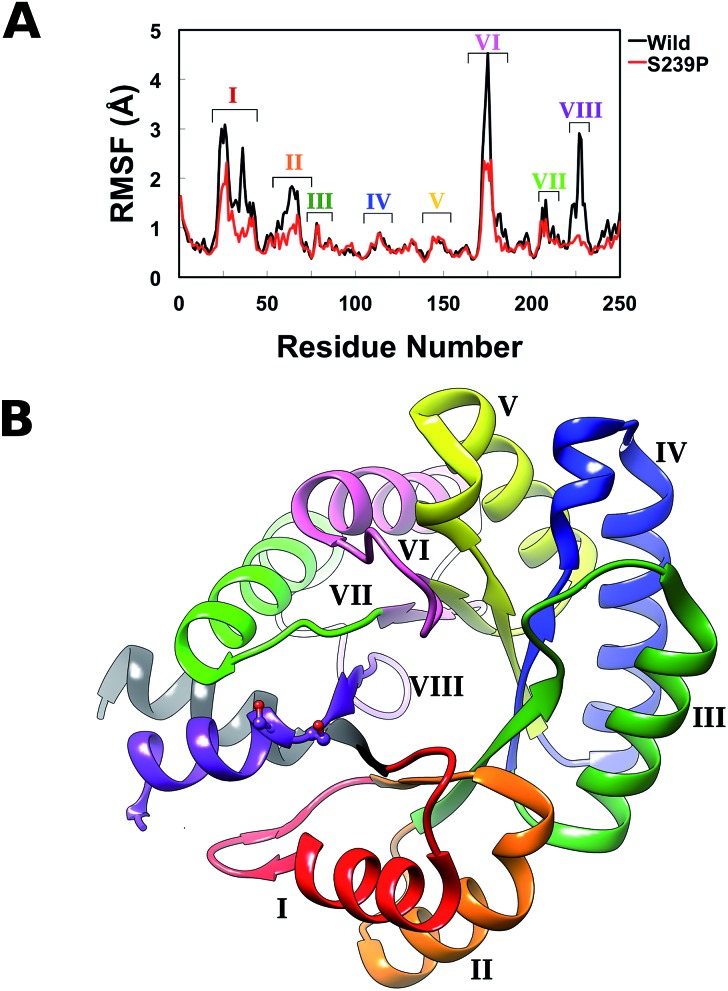
(A) Average root mean square fluctuations, averaged over all three MD trajectories for wild-type DERA and the S239P mutant. The key peaks on the RMSF plot are colour coded on the protein in panel (B).

Finally, in order to further explore the effect of mutations at positions 238 and 239 on overall structural stability, we performed molecular dynamics (MD) simulations of both wild-type DERA, and the S238P, S239P, S238P/S239P and S238I/S239I mutants in their substrate free forms. The simulation setup was as described in the Experimental section, and each variant was equilibrated for a total of 300 ns over 3 replicas with different initial velocities (100 ns each), leading to a total of 1.5 μs of cumulative simulation time for all variants. Fig. S4† shows the root mean square deviations of the backbone atoms for each variant, averaged over 3 replicas, demonstrating the convergence of the simulations. Following from this, we calculated root mean square fluctuations (RMSF) along the MD trajectories for each individual variant (Fig. 5), as well as calculating dynamic cross correlation maps (DCCM) from the MD trajectories, using the Bio3D package.^29^,^30^

**Fig. 6:**
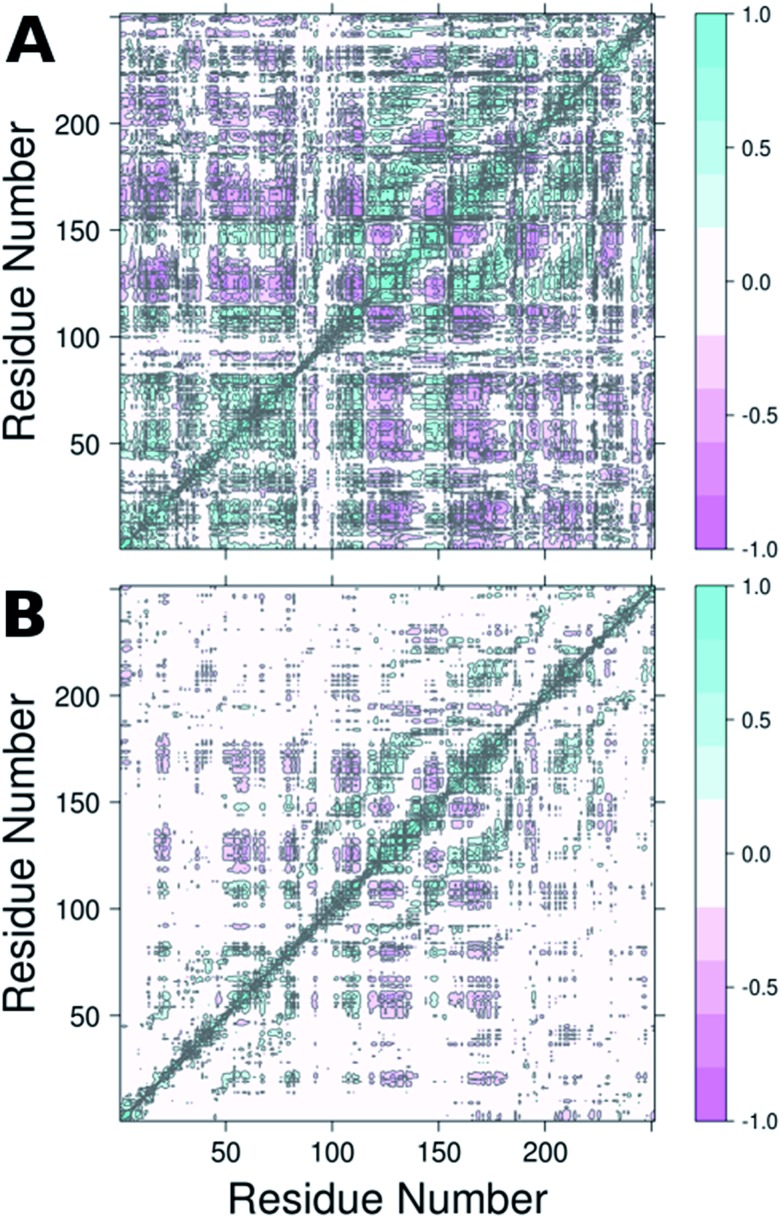
Dynamic cross correlation maps (DCCM) of (A) wild-type DERA and (B) the S239P mutant. Corresponding DCCM plots for all mutants can be found in Fig. S5.† This figure was generated using Bio3D.^29^,^30^

From the RMSF plot, it can be seen that the enzyme is fairly flexible, with each peak on the RMSF plots corresponding to a different helix in DERA (see Fig. 5 for a colour coded comparison of the different helices of DERA and each peak on the RMSF plot), thus corresponding to “breathing motions” of this enzyme. As can be seen from these plots, the introduction of all mutants dampens the flexibility of the enzyme in several regions, and, in particular, the S239P mutant shows less flexibility around residues 25–75, 170–180 and 220–230 (see Fig. 5 for colouring). Following from this, a side-by-side comparison of the DCCM plots for the wild-type and S239P type mutant forms of DERA (Fig. 6) show that this substitution leads to a substantial loss in, in particular, anti-correlated motions. Interestingly, if one then extends this to placing all variants shown in Table 1 side by side, and ranking them according to their measured *k*_cat_/*K*_M_ values, it can be seen that this loss of anti-correlated motions follows a clear trend across the mutants, with the variants with the lowest *k*_cat_/*K*_M_ values (*i.e.* S238P and the S238P/S239P and S238I/S239I double mutants) showing much lower correlated motions (Fig. S5†). This is in good agreement with previous work that suggested the presence of coupled networks of residues in enzymes that play a role in enzyme function (*e.g.*ref. 10–17 among many others), and in particular work by Bruice which argues for anti-correlated motions as a driving force in catalysis.^31^ Therefore, the introduction of the mutations at positions 238 and 239 appears to alter the enzyme's dynamics, rigidifying it, which will in turn lead it to sample fewer catalytically active conformations and impair both substrate binding and subsequent TS stabilization, as shown also in the trends in *k*_cat_/*K*_M_ in Table 1.

DERA's requirement for phosphorylated substrates can be due to different reasons. (1) The obvious, and most trivial reason is to function as a negatively charged anchor binding to a complementary binding site, thereby increasing the affinity for the substrate in the ground state and throughout the reaction. This could be argued as being particularly important for small substrate molecules such as G3P. Any contribution to substrate selectivity, however, is questionable since the relatively strong binding interactions between the protein and phosphate moiety may overshadow the weaker (*e.g.* hydrogen bonding) interactions that steers substrate selectivity, including stereoselectivity. (2) Extensive experimental work by Richard and co-workers on other enzymes acting on phosphorylated substrates, which include orotidine monophosphate decarboxylase^32^–^34^ and triosephosphate isomerase,^35^–^37^ shows that remote protein–phosphodianion interactions can, in these cases, be crucial for transition state stabilization, in part by promoting the formation of (and stabilizing of) active high-energy conformations of the protein. (3) In the current case of DERA catalysed aldol cleavage of dR5P, the additional ring opening of the predominantly existing furanose form (99.8%^38^) of the aldose substrate to the reactive open-chain aldehyde has to be considered (the concentration of the aldehyde in solution is expected to be even lower than 0.2% of the total dR5P, since it will be further equilibrated with the hydrated *gem*-diol). The phosphate group on the furanose may in this case act as an affinity tag facilitating imperfect binding to the active site in which subsequent ring opening is catalysed. The ring opening can be facilitated both by acid catalysis but also by allowing for new binding interactions once the sugar is in the open-chain form, and the aldehyde thus formed is trapped as the imine intermediate after attack by Lys^167^.

For comparison, note that phosphoglucose isomerase, which catalyses the isomerization of glucose-1-phosphate and fructose-1-phosphate faces the same problem. This enzyme acts on the open chain of the aldose or ketose substrates, while these sugars are present in the respective pyranose and furanose forms in aqueous solution, to an even higher degree than in dR5P. Crystal structures have been solved in which both the cyclized and the open-chain form of the substrates can be observed within the same active site. In this enzyme, a His residue has been suggested to be the acid catalyst for sugar ring opening once bound within the active site.^39^ There is no residue in DERA that corresponds to the catalytic His residue present in phosphoglucose isomerase, but rather, there is a strategically positioned water molecule aligned in a hydrogen-bonding network involving the O4 of the open-chain Schiff base and the ammonium of Lys^172^ (PDB ID: ; 1JCJ 7,8), suggesting possible acid functionality. The uncatalyzed ring-opening of furanoses is comparably slow, with rates well below 1 s^–1^.^40^,^41^ This is at least 15-fold below the value of *k*_cat_ for cleavage of dR5P by DERA (Table 1). Hence, catalysed ring-opening must exceed at least the value of the turnover number. Phosphorylation, however, considerably increases the ring-opening rate (40–100 s^–1^for R5P, depending on anomer).^42^ The ring-opening of dR5P is therefore not expected to be rate-limiting to any extent. The aldehyde thus formed is trapped as the imine intermediate after attack by Lys^167^.

## Conclusions

In conclusion, in the present work, we have performed a combined experimental and computational study of the effect of mutagenesis of Ser^238^ and Ser^239^ on the structural dynamics and resulting phosphodianion stabilization of DERA. We demonstrate that although site-saturation mutagenesis suggests that the specific presence of a Ser at these positions is less important for the catalytic activity of the enzyme (as other residues still showed some activity), nevertheless, by substituting Ser for Pro at these positions, we dampen the dynamics of the enzyme with a concomitant loss in both TS entropy and catalytic activity. Additionally, the structural fluctuations of the wild-type enzyme appear to be highly (anti-) correlated, and the loss of catalytic activity appears to be directly connected to the loss of these correlated motions. Therefore, we demonstrate here a critical role for the interplay between coupled motions, entropic changes and flexibility in driving the catalytic activity of this enzyme, and postulate that these features can be regulated for targeted engineering of DERA to accept new substrates.

## Experimental

A detailed description of the experimental is given in the ESI,† and also described briefly below.

### Enzyme production, mutagenesis and kinetic characterization

The coding region of the DERA gene was obtained by colony PCR from *E. coli* XL1-Blue, and the amplified fragment was inserted into the pGT7 expression vector.^43^ Mutations were introduced by overlap mutagenesis PCR with the wild-type DERA gene as template. See Table S1† for oligonucleotide sequences. DERA genes, wild-type and mutants, were transformed into *E. coli* BL21-AI (Invitrogen) for protein expression. Screening of the NDT-mutated gene library for expression of DERA variants with aldol addition activity with acetaldehyde and phenyl acetaldehyde was performed in 96-well plate format, essentially as described in ref. 44, but with modifications for the current expression vector and host cell. Aldolase activity upon incorporating ^14^C-labeled acetaldehyde was detected after separation by thin layer chromatography followed by autoradiography, of the reaction mixtures after incubation of DERA-variant expressing bacterial lysate with labelled and cold acetaldehyde and phenyl acetaldehyde (Fig. S2†).

Purification of DERA variants was accomplished through nickel IMAC, as previously described.^43^ The purified protein was stored in 50 mM triethanolamine buffer, pH 8.0. The rates of catalysed aldol cleavage of dR5P were assayed as described in ref. 45, and steady state kinetic parameters were determined after fitting the Michaelis–Menten equation to the data using SIMFIT (; http://www.simfit.org.uk). In addition, catalyzed rates, under saturating (*k*_cat_) or unsaturating (*k*_cat_/*K*_M_) conditions, in the presence of wild-type DERA or the S239P mutant, were determined at different temperatures (15 °C, 20 °C, 25 °C, 30 °C, 38 °C and 45 °C). The natural logarithms of the ratios of the determined rates were plotted as functions of the inverse of *T* to allow for extraction of the changes in transition state enthalpy and entropy (eqn (1)), caused by the S239P mutation.1
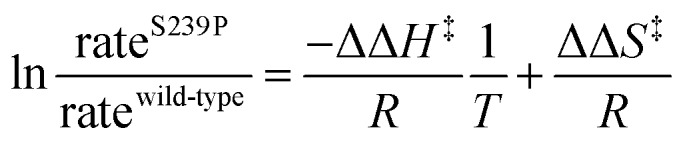



The steady state kinetic parameters for wild-type DERA, and the S239P and S238I/S239I mutants were also determined at pH values of 7, 8 and 9. The results are presented in Fig. S1 and Table S2.†

### Computational approaches

All molecular dynamics simulations of DERA were performed using the Q simulation package^46^ and the OPLS-AA force field.^47^ The 0.99 Å resolution structure of wild-type DERA (PDB ID: ; 1P1X 8,9) was used as the starting point for all simulations, and the relevant mutations were introduced to this structure using Richardson's common-atom rotamer library^48^ as implemented in the UCSF Chimera software package. In each case, the system was solvated using a 35 Å sphere of TIP3P water molecules, with a 10 kcal mol^–1^ Å^–2^ positional restraint on the 15% outer layer of the solvent sphere to stabilize it during the simulation, which is necessary using spherical boundary conditions (see also *e.g.* our recent work^49^,^50^). All residues (apart from the Schiff base) were protonated according to their preferred protonation state at physiological pH, resulting in an overall system charge of –5, which was neutralized with Na^+^ counterions. After initial solvation, the system was gradually heated from 0 to 300 K over the course of 1 ns using a 1 fs timestep and the Berendsen thermostat.^51^ The structure was subject to an initial positional restraint of 200 kcal mol^–1^ Å^–2^ on all heavy atoms to allow protons to equilibrate to their optimal positions and remove initial bad contacts in the crystal structure, and this restraint was gradually dropped during the heating procedure, as the structure was allowed to relax. After this initial equilibration, we ran a further 100 ns production run, using 3 replicas for each system with different initial velocities (*i.e.* 300 ns simulation time per system) The only restraint kept in the actual production run was a weak 3 kcal mol^–1^ Å^–2^ positional restraint on the Na^+^ counterions to keep them within the simulation sphere. This procedure was performed on both the wild-type enzyme as well as the S238P, S239P, S238P/S239P and S238I/S239I mutants, and the final 100 ns of each trajectory were retained for further analysis. All analysis was performed externally by importing our Q trajectories into GROMACS,^52^ with the exception of the dynamic cross correlation analysis, which was performed using the Bio3D package.^29^,^30^

## Supplementary Material

Supplementary information
