# Supplementary material for: Linking coupled motions and entropic effects to the catalytic activity of 2-deoxyribose-5-phosphate aldolase (DERA)
Source: Chem Sci. 2015 Nov 17;7(2):1415–21. doi: 10.1039/c5sc03666f (PMC5975929; doi:10.1039/c5sc03666f)
Supplement: Supplementary file 1 [file SC-007-C5SC03666F-s001.pdf]

Electronic Supplementary Material (ESI) for Chemical Science.
This journal is © The Royal Society of Chemistry 2015

Supplementary Information for:

Linking Coupled Motions and Entropic Effects to the Catalytic Activity of 2-deoxyribose-5-phosphate Aldolase (DERA)

Huan Ma,[a] Klaudia Szeler,[b] Shina C. L. Kamerlin[b,*] and Mikael Widersten[a,*]

[a] Department of Chemistry – BMC, Uppsala University, Box 756, SE-751 23, Uppsala, Sweden.

[b] Department of Cell and Molecular Biology, Uppsala University, Box 596, SE-751, Uppsala, Sweden

kamerlin@icm.uu.se, mikael.widersten@kemi.uu.se

Table of Contents

Experiments and methods……………………………………………………………………………………………………S2

Chemicals, nucleotides and reagents……………………………………………………………………………………………S2

Cloning of the *E. coli* DERA gene……………………………………………………………………………………………….S2

Construction of the S238P, S239P and S238P/S239P DERA mutants……………………………………………………….S2

Construction of the S238/S239 DERA mutant library……………………………………………………………………………S2

Screening of the mutant library for DERA variants showing phenyl acetaldehyde activity…………………………S3

Activity screening with ^{14}C-labeled acetaldehyde……………………………………………………………………………S3

Expression and purification of *E. coli* DERA variants……………………………………………………………………….S4

Kinetic characterization……….S5

Effects of the S238P mutation on reaction thermodynamics……………………………………………………………….S5

pH-dependence of catalysis……………………………………………………………………………………………………….S5

Figures and Tables………S6

Figure S1. pH dependence of the catalytic rates of the wild-type, S238P and the S238I/S239I DERA variants………S6

Figure S2. Autoradiograph of TLC-separate reaction mixtures of ^{14}C-labeled acetaldehyde and phenyl acetaldehyde catalyzed by DERA variants……………………………………………………………………………….S7

Figure S3. Amino acids at positions 238 and 239 in scored hits after sceening for aldol addition activity with ^{14}C-labeled acetaldehyde and phenyl acetaldehyde…………………………………………………………………S7

Figure S4. C_{α} RMSD plots for wild-type DERA, as well as the S238P, S239P, S238P/S239P and S238I/S239I mutant forms of the enzyme……………………………………………………………………………………………….S8

Figure S5. Dynamic cross-correlation maps for wild-type DERA as well as the S238P, S239P, S238P/S239P and S238I/S239I mutant forms of the enzyme………………………………………………………………………….S9

Figure S6. Temperature stability of wild-type DERA……………………………………………………………………S10

Table S1. Oligonucleotide sequences used for site-directed mutagenesis……………………………………….S11

Table S2. Steady-state parameters for aldol cleavage of dR5P, determined at pH 7-9……………………………S12

Table S3. Steady-state parameters for aldol cleavage of dR5P, determined at different temperatures…….S13

Experiments and Methods

Chemicals, nucleotides and reagents. Synthetic oligonucleotides were purchased from Thermo Fisher Scientific GmbH (Ulm, Germany). All restriction enzymes, Taq DNA polymerase and supplement chemicals and buffers for molecular cloning were purchased from Thermo Fisher Scientific if not otherwise stated. The bacterial culture media components tryptone and sodium chloride were purchased from Sigma-Aldrich, and Merck supplied agar and yeast extract. Nickel (II) Sepharose 6 Fast Flow gel for IMAC purification and a disposable PD-10 desalting column were purchased form GE Healthcare Life Science. All the other chemicals, antibiotics, buffer components and enzymes were purchased from Sigma-Aldrich if not otherwise stated. Standard procedures and reaction conditions were used for gene cloning, and host cell transformations were performed as stated in the user manual from the manufacturers. DNA sequencing services were provided by the Uppsala Genome Center (Uppsala, Sweden).

Cloning of the *Escherichia coli* DERA (2-deoxyribose-5-phosphate aldolase) gene. The DERA gene was obtained by colony PCR with the following pair of cloning primers: *DERA-Fwd,* 5'-TTT TTT <u>CTC GAG</u> ATG ACT GAT CTG AAA GCA AGC-3', and *DERA-Rev*, 5'-TTT TTT <u>ACT AGT</u> GTA GCT GCT GGC GCT CTT AC-3'. The amplified region corresponded to base pairs 1 to 780 in the 2-deoxyribose-5-phosphate aldolase (ECs5340) gene of *E. coli* strain TB182A (GenBank accession number EU890178, ID: ACI72764.1). The underlined sequences indicate restriction sites for *Xho*I and *Spe*I, which were introduced at the 5'- and 3'-ends, respectively, of the amplified gene fragement. Cells from the *E. coli* strain XL1-Blue (Strategene Corp.) were used in the colony PCR. The resulting 780 bp DERA DNA fragment was gel purified and its sequence confirmed. The DNA fragment was subsequently digested with *Xho*I and *Spe*I and ligated into the pGT7 expression vector[1]. The vector introduces codons for a C-terminal $(His)_5$-Tag. The ligated construct was transformed into *E. coli* XL1-Blue. The integrity of the resulting plasmids were verified by restriction analysis and by DNA sequencing.

Construction of the DERA S238P, S239P and S238P/S239P mutants. Mutations were introduced by overlap mutagenesis PCR with the wild-type DERA gene as template. The oligonucleotide sequences used in PCR are given in Table S1. The mutameric oligonucleotides allows for isolation of either of the single mutants and the double mutant from the same mixture of amplified fragments. The identities of the mutated fragments were confirmed by sequencing, and the different gene fragments were individually sub-cloned into pGT7, as described for the wild-type gene fragment.

Construction of the DERA S238/S239 mutant library. Random mutations were introduced at codons 238 and 239 by overlapping PCRs with mutagenesis primers containing the NDT degenerate codon mix of 12 codons using the wild-type DERA gene as template. The NDT codon set encodes for F, L, I, V, Y, H, N, D, C, R,

S and G. The encoded amino acid residues contribute difference side chain properties and reactivities. Table S1 shows the sequences of the oligonucleotides used in the mutagenesis PCRs. The library gene fragments were sub-cloned as described for the wild-type DERA gene.

Screening of the mutant library for DERA variants showing activity with phenyl acetaldehyde as the acceptor substrate. The S238X, S239X mutant gene library was transformed into *E. coli* BL21-AI (Invitrogen) and plated on agar plates fortified with 100 µg/ml ampicillin and grown overnight. Single colonies were randomly picked and inoculated into 96-well plates (500 µl wells, VWR). Clones expressing wild-type DERA or an alcohol dehydrogenase A (ADH-A) were also picked as negative controls. The cells were grown in 350 µl 2TY containing 100µg/ml ampicillin. The plates were sealed with gas-permeable adhesive film and incubated at 30 °C overnight with shaking at 150 rpm. 25 µl of the overnight cultures were used to inoculate 325 µl 2TY (with 50µg/ml ampicillin) to form new cultures for protein expression. The cultures were incubated at 30 °C for approximately 4 h until OD_{600} ~0.8. At this point, 3.5µl of 20 % L-arabinose solution was added (0.2 %, w/v, final concentration) for induction of protein expression. The expression cultures were grown for an additional 3 h, and the cells were then harvested by centrifugation at 2191×g for 20 min at 4 °C. The supernatants were discarded. The 96-well plates containing the cell pellets were stored at -80 °C until screening for aldolase activity. 55 µl of autoclaved 87 % (v/v) glycerol was added into the remaining 325µl of overnight cultures, and these plates were stored at -80 °C for backup.

Activity screening with ^{14}C-labeled acetaldehyde.

Cell pellets were resuspended in 100 µl triethylamine buffer (50 mM TEA, pH 8.0) with complete EDTA-free protease inhibitor tablets added (Roche) and 25 µl B-PER (Bacteria Protein Extraction Buffer, Thermo Scientific). The plates were incubated for 45 min at room temperature, with shaking on a plate vortex. The plates were then centrifuged at 2191×g for 1 h at 4 °C. 75 µl clear lysate from each well was collected for activity screening.

Wild-type DERA displays no detectable aldol addition activity with phenyl acetaldehyde as the acceptor substrate. This compound was therefore considered to be a good candidate substrate, as it both lacks a phosphate group *and* instead contains a non-polar benzyl substituent. Since *any* possible activity that could have arisen in some DERA library variant was expected to be low, an activity screen that provided high sensitivity was designed. Hence, the screening assay for aldol addition of acetaldehyde and phenyl acetaldehyde was based on separation of the aldol product(s) by thin layer chromatography (TLC) after incorporation of radioactive ^{14}C-labeled acetaldehyde followed by autoradiography (Fig. S2).

Acetaldehyde (1,2-^{14}C) was ordered from Larodan Fine Chemicals AB (Stockholm, Sweden). The concentration was 0.6 mCi/ml (60 mM acetaldehyde) with a specific activity of 10 mCi/mmol. The screening

was performed as follows: 45 µl cell lysate from each well was mixed with 4 µl of 0.05 mM acetaldehyde, 2 µl of 1.3 M phenyl acetaldehyde, dissolved in acetonitrile (26 mM final concentration), 10 µl 0.06 mCi/mL ^{14}C-labeled acetaldehyde and 39 µl of 50 mM TEA pH 8.0 buffer. The reaction mixture was incubated at 30 °C for 4 h with 100 rpm shaking. Lysates from wild-type DERA, ADH-A or protein extraction buffer only, were included in control reactions. 10 µl from each reaction mixture was spotted on silica gel on TLC AL foils (Fluka). The mobile phase for TLC was ethylacetate:pentane, 1:1(v/v). Air-dried TLC plates were then positioned to face the emulsion side of Kodak BioMax MR films (Sigma-Aldrich). The films were exposed overnight on a cassette in the dark, and developed according to the instructions from the manufacturer. The developed films were then scanned for further quantification analysis. Fig. S2 shows a typical result from such a screen.

The expected product from aldol addition is 3-hydroxy-4-phenylbutanal, which contains both a hydroxyl group and an aldehyde group, and therefore its polarity is expected to be between that of phenyl acetaldehyde and 1-phenyl-1,2-ethanediol. Furthermore, in aqueous solution, any aldol product formed will be present in both its aldehyde and its hydrated *gem*-diol forms, where the *gem*-diol will be the more polar. Based on TLC results, using phenyl acetaldehyde and 1-phenyl-1,2-ethanediol as reference compounds, we assumed that the spot migrating faster and closer to the solvent front is the aldol product between phenyl acetaldehyde and acetaldehyde (considering also that the *gem*-diol form of 3-hydroxy-4-phenylbutanal will be more polar and has an even lower R_f value than the aldehyde). The intensity of a signal spot on the film is proportional to the amount of ^{14}C-labeled acetaldehyde incorporated. Based on these two assumptions, the intensity of the signal spots were analyzed by pixel counting using ImageJ (http://imagej.nih.gov/ij/). The intensity of each signal spot was normalized in order to minimize the random error, and enzyme variants that had been present in reactions resulting in increased intensities in the faster migrating spots after TLC were scored as hits.

Expression and purification of *E. coli* DERA variants. The pGT7-DERA-5His plasmid containing the wild-type or mutated DERA gene was transformed into *E. coli* BL21-AI strain for protein expression. All transformation and expression steps were carried out according to the user manual from manufacturer. In brief, 10 ml overnight cultures were used to inoculate 1 L of 2TY (50µg/ml ampicillin). The cultures were grown at 30 °C until OD_{600} of ~0.8 when L-arabinose was added to 0.2 % (w/v). Protein expression was continued for 3 h. Cells were harvested by centrifugation at 3024×*g* for 15min at 4°C. The cell pellet was resuspended in IMAC Binding Buffer (10 mM sodium-phosphate, pH 7.5, 500 mM NaCl, 20 mM imidazole). The resuspended cells were lysed by passing the mixture through a French-press cell disruptor. The lysate was subsequently centrifuged at 15,000×g for 40 min. The cleared supernatant was loaded onto a pre-equilibrated Ni (II)-IMAC column of 2 ml Ni-Sepharose 6 Fast Flow gel. The column was washed twice with

15 ml Washing Buffer (same composition as Binding Buffer but containing 100 mM imidazole). After washing, bound protein was eluted by addition of 2.5 ml Elution Buffer twice (same composition as Binding Buffer but containing 300 mM imidazole). The protein solution obtained was desalted by passing through a PD10 column equilibrated with 50 mM TEA buffer, pH 8.0. The purity of the protein was confirmed by SDS-PAGE stained with Coomassie Brilliant Blue R-250, as well as by quantitative amino acid analysis. The extinction coefficient of DERA at 280 nm was determined to be 17,548 $M^{-1}\,cm^{-1}$.

Kinetic characterization. The steady-state kinetic parameters of both wild-type and mutant DERA variants were determined by a previously described method[2]. All steady-state kinetic measurements were carried out in 50 mM TEA buffer, pH 8.0, 30 °C, at increasing concentrations of dR5P and in the presence of saturating concentration of NADH (0.5 mM) and an excess (0.14 units) of α-glycerophosphate dehydrogenase-triosephosphate isomerase coupled-enzyme mix. The Michaelis Menten equation was fitted to the raw data using MMFIT in SIMFIT (http://www.simfit.org.uk/) to determine V_{max} and $K_{\mathrm{M}}^{\mathrm{dR5P}}$. $V_{\mathrm{max}}/K_{\mathrm{M}}$ was determined by fitting Eq. **1** to the raw data using RFFIT in SIMFIT.

$$v_0 = \frac{V_{\max} / K_M [S]}{1 + [S] / K_M} \tag{1}$$

Effect of the S239P mutation on reaction thermodyamics. Catalyzed rates, under saturating (k_{cat}) or unsaturating ($k_{\mathrm{cat}}/K_{\mathrm{M}}$) conditions, in the presence of wild-type DERA or the S239P mutant, were determined at different temperatures (15 °C, 20 °C, 25 °C, 30 °C, 38 °C and 45 °C, with the results illustrated in Figs. S6 and Fig. 4 of the main text). The natural logarithms of the ratios of the determined rates were plotted as functions of the inverse of T to allow for extraction of the changes in transition state enthalpy and entropy (Eq. 2), caused by the S239P mutation.

$$\ln \frac{\mathrm{rate}^{\mathrm{S239P}}}{\mathrm{rate}^{\mathrm{wild\ type}}} = \frac{-\Delta\Delta H^{\ddagger}}{R} \cdot \frac{1}{T} + \frac{\Delta\Delta S^{\ddagger}}{R} \tag{2}$$

pH-Dependence of Catalysis. Steady-state kinetic parameters for wild-type DERA, and the S239P and S238I/S239I mutants were determined at pH values of 7, 8 and 9. The results are illustrated and tabulated in Fig. S1 and Table S2 respectively.

Figures and Tables

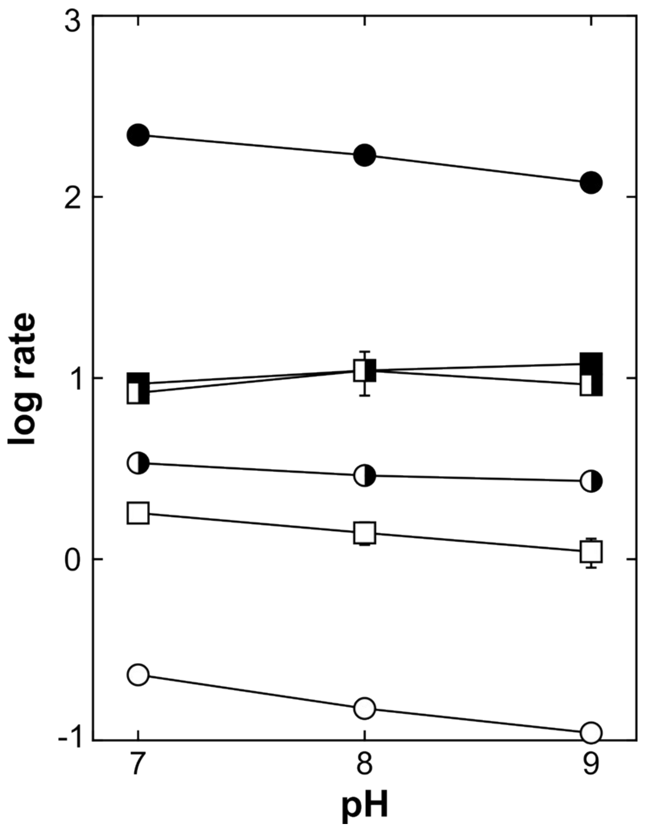

Figure S1. The pH dependence of rates, k_{cat} (squares) and k_{cat}/K_M (circles) in the retro-aldol cleavage of dR5P catalyzed by wild-type DERA (filled symbols), the S239P mutant (half-filled symbols) and the S238I/S239I mutant (unfilled symbols). The effect of pH on catalysis is weak in the tested region, suggesting that the pK_a of the active-site Lys[167] is unchanged by the mutations we introduced. Error bars indicate standard errors over all replicates.

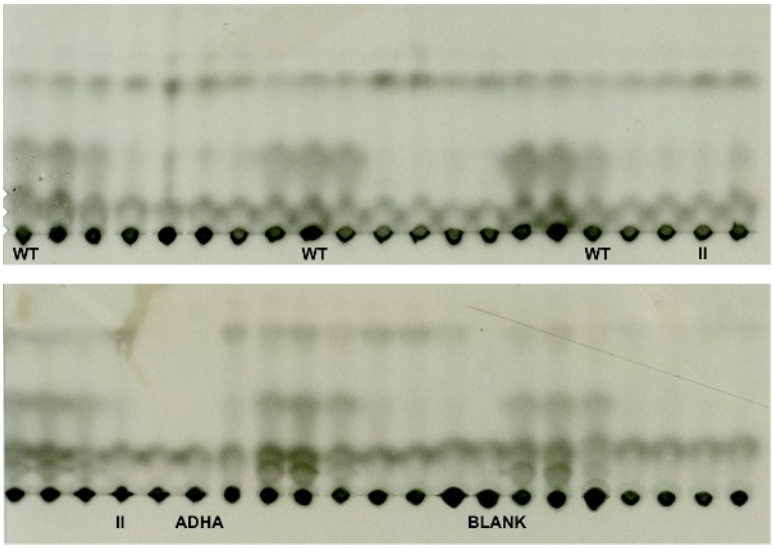

Figure S2. Autoradiograph of the TLC-separated reaction mixtures of ^{14}C-labeled acetaldehyde and phenyl acetaldehyde, catalyzed by DERA variants. The radioactivity intensity of the fastest moving product (at the top of each panel) was used to distinguish hits from the background, applying a threshold of a >2-fold increase over wild-type DERA.

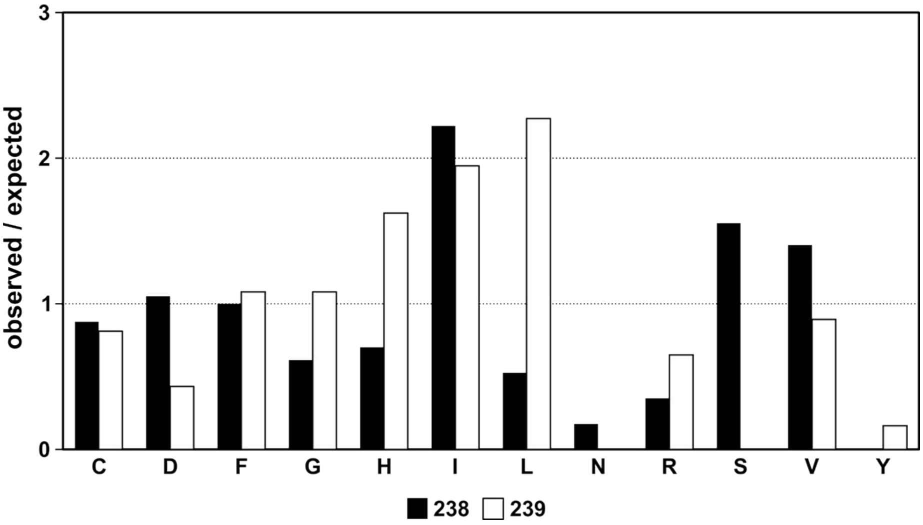

Figure S3. Amino acid residues at positions 238 (filled bars) and 239 (unfilled bars) in scored hits after screening for aldol addition activity with ^{14}C-labeled acetaldehyde and phenyl acetaldehyde. The bar height is normalized for the expected occurrence of a certain residue, as judged from sequencing of clones in the unselected (naïve) library. Ile, Ser and Val are over-represented at position 238 and Leu, Ile and His at position 239. It is noteworthy to observe that Ser is counter-selected for at position 239 in these DERA variants.

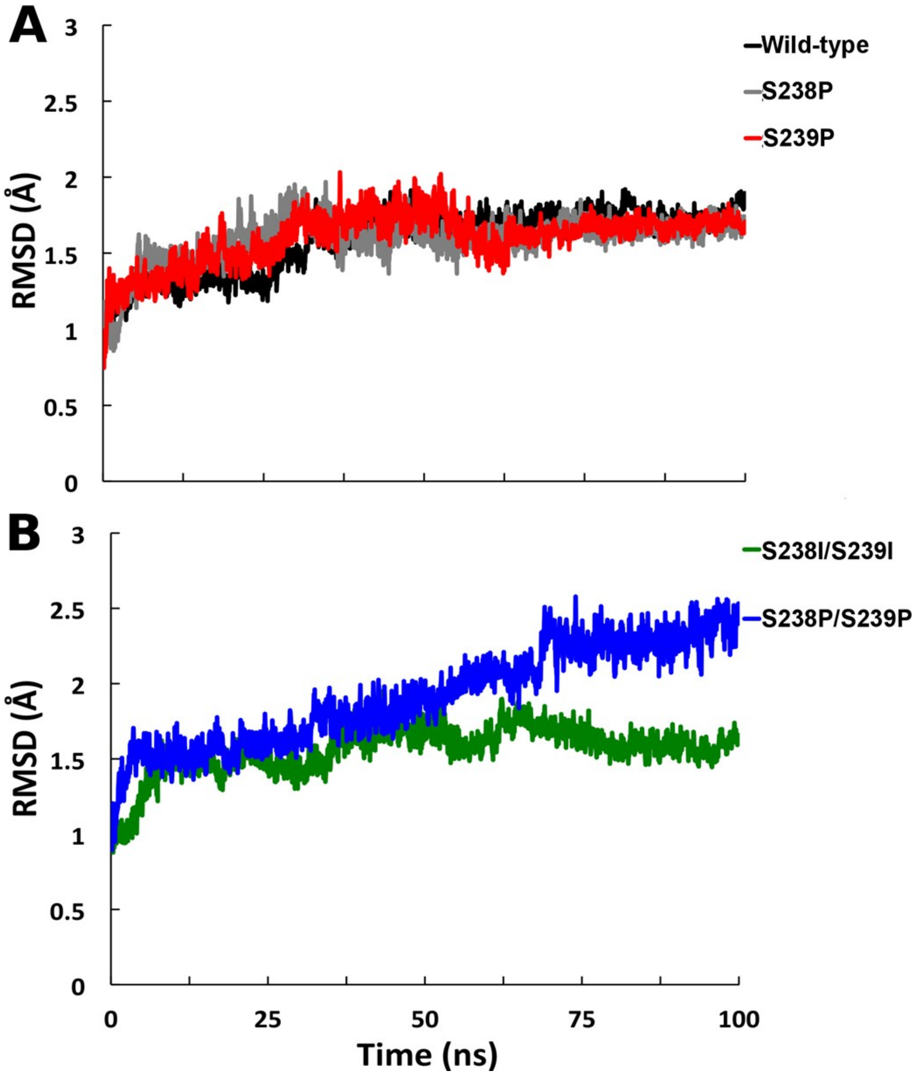

Figure S4. C_{α} RMSD plots for (A) wild-type DERA and the S238P and S239P single mutants, and (B) the S238P/S239P and S238I/S239I double mutants. These plots are averages over three trajectories. As can be seen from panel (B), even after 100ns, the S238P/S239P double mutant has not converged, and this structure is inherently unstable. From Table 1 in the main text, it can be seen that the catalytic ability of this variant is abolished upon introducing this mutation, and from Fig. S5 it can be seen that there is a significant reduction of correlated motions in this enzyme variant, suggesting that this mutation substantially perturbs the enzyme's dynamical behaviour in a negative way.

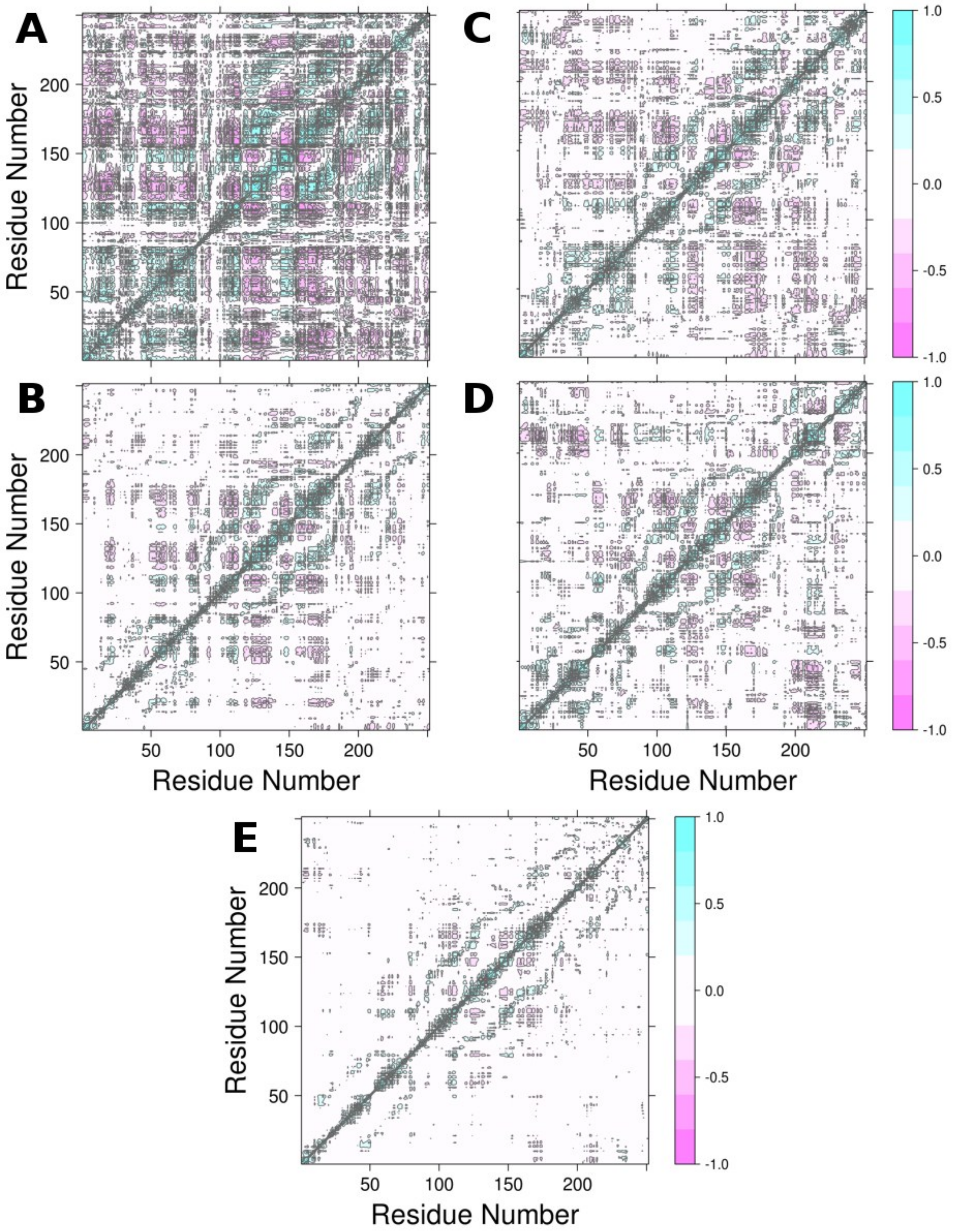

Figure S5. Dynamic cross correlation maps (DCCM) for (A) wild-type DERA, and the (B) S239P, (C) S238I/S239I, (D) S238P/S239P and (E) S238P mutant forms, ranked according to the k_{cat}/K_M values provided in Table 1 of the main text. All DCCM were calculated using Bio3D[3, 4], and are averages over 3 trajectories.

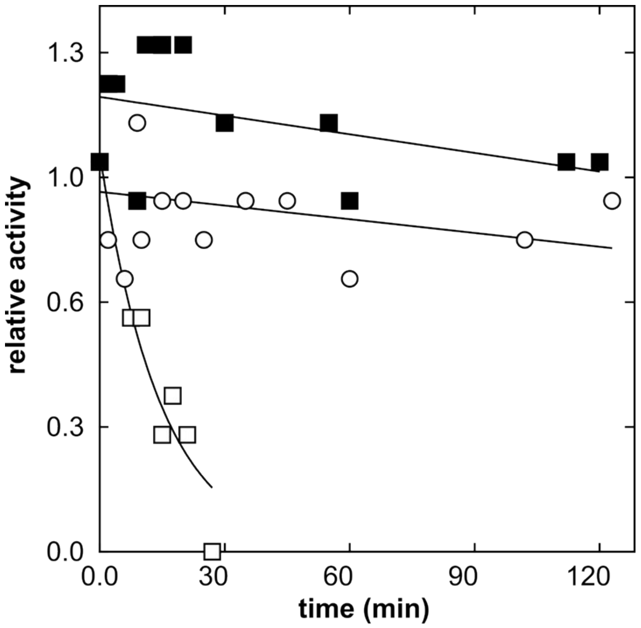

Figure S6. Temperature stability of wild-type DERA (filled symbols) and the S239P mutant (hollow symbols). The enzymes were incubated at 45 °C (circles) or 60 °C (squares) and aliquots were removed for assaying remaining activity dR5P cleavage. Wild-type DERA remains fully active >2 h incubation at 60 °C, whereas the S239P mutant looses activity within 30 min at the same temperature, demonstrating a lower stability of the mutant. The assay was performed as described in the Experimental section.

Table S1. Oligonucleotide sequences used for site-directed mutagenesis and for construction of the mutant-library carrying NDT codons at positions 238 and 239[a].

Label	Sequence 5'→3'
DERA-Fwd	TTT TTT CTC GAG ATG ACT GAT CTG AAA GCA AGC
DERA-Rev	TTT TTT ACT AGT GTA GCT GCT GGC GCT CTT AC
DERA P1	CGT CAC TAC CGC TTT GGT GCT **Y**CC **Y**CC CTG CTG GCA AGC CTG CTG
DERA P2	AGC ACC AAA GCG GTA GTG ACG
DERA P3	CGT CAC TAC CGC TTT GGT GCT **NDT NDT** CTG CTG GCA AGC CTG CTG

[a] The underlined sequences indicate restriction enzyme recognition sites. The bold letters **Y** indicate mutated sites, allowing for insertion of either T (resulting in a silent mutation) or C (resulting in a Ser→Pro mutation) in the amplified gene fragments. The **NDT** codons indicate positions mutated for construction of mutant-library.

Table S2. Steady-state parameters for aldol cleavage of dR5P, determined at pH 7-9.

DERA variant	k_{cat} (s^{-1})	K_M (M)	k_{cat}/K_M (s^{-1} M^{-1})
**pH 7**			
**wild type**	9.3±0.2	(4.3±0.3) x 10^{-5}	(2.2±1) x 10^{5}
**S239P**	8.3±0.4	(2.4±0.2) x 10^{-3}	(3.4±0.07) x 10^{3}
**S238I/S239I**	1.8±0.1	(7.6±0.7) x 10^{-3}	(2.3±0.07) x 10^{3}
**pH 8**			
**wild type**	11±0.5	(6.1±0.7) x 10^{-5}	(1.7±1) x 10^{5}
**S239P**	11±3	(3.5±1) x 10^{-3}	(2.9±0.2) x 10^{3}
**S238I/S239I**	1.4±0.2	(9.6±1) x 10^{-3}	(1.5±0.06) x 10^{2}
**pH 9**			
**wild type**	12±0.6	(1.1±0.2) x 10^{-4}	(1.2±0.6) x 10^{5}
**S239P**	9.2±0.8	(3.4±0.4) x 10^{-3}	(2.7±0.08) x 10^{3}
**S238I/S239I**	1.1±0.2	(1.0±0.2) x 10^{-2}	(1.1±0.05) x 10^{2}

Table S3. Wild-type or S239P catalyzed steady-state rates for aldol cleavage of dR5P at different temperatures.

DERA Variant	T (°C) 15	20	25	30	38	45
k_{cat} (s^{-1})						
**wild type**	8.0±0.4	9.3±0.4	14±0.05	15±0.4	20±0.2	26±0.1
**S239P**	5.7±0.5	8.6±0.6	12±0.7	11±1	14±0.08	15±0.8
k_{cat}/K_M (s^{-1} M^{-1})						
**wild type**	(5.3±0.3) x 10^4	(7.6±0.4) x 10^4	(8.8±0.3) x 10^4	(1.3±0.07) x 10^5	(3.0±0.2) x 10^4	(4.8±0.02) x 10^4
**S239P**	(1.8±0.08) x 10^3	(1.8±0.03) x 10^3	(2.6±0.04) x 10^3	(3.6±0.06) x 10^3	(5.5±0.2) x 10^3	(7.8±0.5) x 10^4

References

1. L. T. Elfström and M. Widersten, *Biochim. Biophys. Acta*, 2005, **1748**, 213-221.
2. G. DeSantis, J. Liu, D. P. Clark, A. Heine, I. A. Wilson and C. H. Wong, *Bioorg. Med. Chem.*, 2003, **11**, 43-52.
3. B. J. Grant, A. P. Rodrigues, K. M. ElSawy, J. A. McCammon and L. S. Caves, *Bioinformatics*, 2006, **22**, 2695-2696.
4. L. Skjærven, X. Q. Yao, G. Scarabelli and B. J. Grant, *BMC Bioinformatics*, 2014, **15**, 399.
